# Improved Tet-responsive promoters with minimized background expression

**DOI:** 10.1186/1472-6750-10-81

**Published:** 2010-11-24

**Authors:** Rainer Loew, Niels Heinz, Mathias Hampf, Hermann Bujard, Manfred Gossen

**Affiliations:** 1 EUFETS GmbH, 55743 Idar-Oberstein, Germany; 2 Zentrum für Molekulare Biologie (ZMBH), 69120 Universität Heidelberg, Heidelberg, Germany; 3 Hannover Medical School, 30625 Hannover, Germany; 4 Max Delbrück Center for Molecular Medicine (MDC), 13125 Berlin, Germany; 5 Berlin-Brandenburg Center for Regenerative Therapies (BCRT), 13353 Berlin, Germany

## Abstract

**Background:**

The performance of the tetracycline controlled transcriptional activation system (Tet system) depends critically on the choice of minimal promoters. They are indispensable to warrant low expression levels with the system turned "off". On the other hand, they must support high level of gene expression in the "on"-state.

**Results:**

In this study, we systematically modified the widely used Cytomegalovirus (CMV) minimal promoter to further minimize background expression, resulting in an improved dynamic expression range. Using both plasmid-based and retroviral gene delivery, our analysis revealed that especially background expression levels could be significantly reduced when compared to previously established "standard" promoter designs. Our results also demonstrate the possibility to fine-tune expression levels in non-clonal cell populations. They also imply differences regarding the requirements for tight regulation and high level induction between transient and stable gene transfer systems.

**Conclusions:**

Until now, our understanding of mammalian transcriptional regulation including promoter architecture is limited. Nevertheless, the partly empirical modification of *cis*-elements as shown in this study can lead to the specific improvement of the performance of minimal promoters. The novel composite Ptet promoters introduced here will further expand the utility of the Tet system.

## Background

The Tet system is the most widely used inducible gene expression technology in eukaryotes, for both, *in vivo *and *in vitro *applications. Based on its original design [1], now frequently referred to as „Tet-Off" system, it experienced its first major modification by the introduction of the „Tet-On" system [2]. Both systems respond in opposite ways to the presence of tetracyclines (e.g. doxycycline, Dox), by either inactivating (Tet-Off) or activating gene expression (Tet-On). The difference is based on amino acid exchanges in the synthetic transcription factors employed, tTA and rtTA respectively, reversing the response of the DNA binding domain to the presence of the allosteric effector Dox. Initially both systems relied on the same composite tet-responsive promoter, Ptet-1 [1]. Subsequent attempts to broaden the utility and performance of the Tet system focused on transactivator manipulation: nuclear localization sequences were introduced [3,4], codon usage was optimized [5-7], potential splice sites were removed [6] and activation domains have been exchanged [8,9]. The most significant advances, however, came from genetic approaches to identify improved versions of Tet-On type transactivators. These experiments aimed to improve the dynamic range, by either a reduction of residual DNA binding (and thus transcriptional activation) in the non-induced state or enhancement of activation in the induced state. In this context, the most notable transactivator alleles were identified in yeast [6] and, through enforced retroviral evolution, in human cells [10,11].

While these approaches focused on improving the dynamic range of the Tet system *via *modified transactivators, it appeared interesting to explore, whether the Tet system could also be improved by manipulation of the *cis*-acting tet-responsive promoters. The initially introduced Ptet-1 [1] is based on a minimal promoter fragment derived from the CMV immediate early promoter. Various alternatives were introduced over the years, like those further truncating the CMV minimal promoter of Ptet-1 [12-15], constructs based on the HSV Tk promoter [1], the MMTV long-terminal repeat promoter [16,17] and the HIV-1 long-terminal repeat promoter [17]. Several of these Ptet variants had their advantages under the specific conditions tested, with e.g. a "second-generation tetracycline regulatable promoter" [13] showing up to 5 orders of magnitude in inducible gene regulation experiments using the Tet-off regulation principle. However, with the exception of Ptet-14 (commonly known as P_tight _[18]), which was derived from Ptet-1 by empirical modifications and displayed a substantially reduced background expression, none of these promoter constructs is widely used.

Here we present our results with a series of novel Ptet variants. Systematically introduced alterations were generated to minimize background expression while maintaining high levels of induced expression. To place these experiments in a proper context, it is worthwhile to initially distinguish between the different sources of such background expression. Especially in a comparative analysis of transient transfection and stable transduction experiments as presented here, their individual contribution can be readily addressed. Considering the various sources of background expression is an essential requirement when expression systems based on the transactivation paradigm are engineered and is not restricted to the Tet system.

*In principle*, background expression can be triggered by residual binding of transactivators to their cognate binding sites. This would be the case when expression, driven by a Ptet promoter, is higher in a tet transactivator-positive cell line in the off-state as compared to an otherwise isogenic tet transactivator-negative cell line.

*Secondly*, background expression due to e.g. enhancers or other sequence elements able to act at long-range on Ptet is to be expected and has indeed been frequently observed. Such effects can be avoided by targeted insertion of the Ptet driven transcription unit into an appropriate genomic locus [19] or suppressed by the use of insulator elements [20].

*Thirdly*, background expression is also influenced by the accessibility of the Ptet, which is determined by epigenetic parameters such as DNA methylation and integration into chromatin structures.

*Lastly*, background expression might be intrinsic for a given Ptet due to hidden binding sites for transcription factor. Reduction of this intrinsic background was the goal of the current study. For this purpose, we designed novel Ptet promoter variants with low background activities and highly induced expression levels by stepwise modification of Ptet-1. Already known functional *cis *elements of the promoter where exchanged, others expected to optimize its performance were introduced and those suspected to impede performance where deleted. This strategy proved successful when the new Ptet promoter variants where quantitatively characterized in a plasmid vs. retrovirus backbone and examined in different cell lines. Our results revealed that several novel Ptet promoters, and in particular, Ptet-T6, are superior to Ptet-1 and Ptet-14 with regard to low background levels in the off-state, while retaining a high activation potential.

## Results

### Experimental strategy

**Figure 1:**
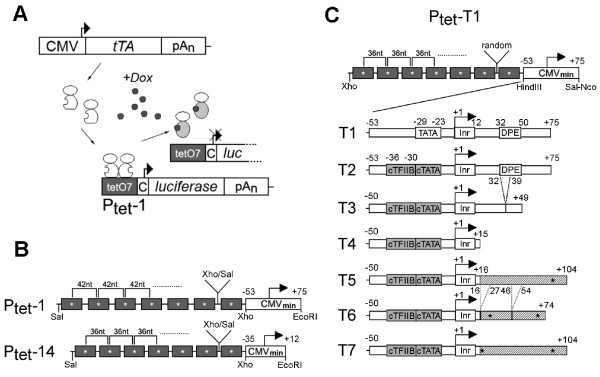
**Outline of the Tet System and the Ptet promoters**. **(A) **The tet-responsive transactivator (here tTA, i.e. Tet-Off system) is constitutively expressed in the cell. tTA homodimers bind to the heptameric tet operator sequence (tetO7) in the absence of Dox (a tetracycline derivative). They dissociate from their recognition sites after Dox addition. The Dox response would be reversed for "Tet-On" type transactivator lines also used in this study (C = CMV minimal promoter). **(B) **Comparison of Ptet-1, the originally described Ptet promoter and the commercially available Ptet-14 ("Ptight"). Both consist of tet operator heptamers created by monomer ligation via compatible restriction sites (Xho/Sal), but with differently spaced operator centers. The CMV-minimal promoters differ in size, but both contain the authentic TATA-box and initiator sequence (Inr). **(C) **The newly designed Ptet promoters are shown, consisting of a tet operator heptamer with 36nt spacing, and randomized fusion points between all the operators. The CMV derived minimal promoters of the T2-T7 Ptet promoters all have a consensus ("c") TATA-box and TFIIB binding site but differ in the composition of the 5' UTR as outlined in the text. Point mutations are indicated by (*), deletions by dashed lines. Nucleotide positions are given relative to the transcriptional start site (+1). The initiator (Inr) and downstream promoter element (DPE) is indicated.

The Tet system with its key elements Ptet and the tetracycline controlled transcriptional activators (tTA and rtTA) is outlined in Figure 1A. Ptet-1 is composed of a CMV derived minimal promoter linked downstream to an array of seven tet operators. The focus of this work was the further optimization of Ptet-1 *via *reduction of background expression while maintaining or even improving the levels of induced expression. Such combined effects would result in an improved range of transcription control.

For comparison Ptet-1 and Ptet-14 were included in the study. As outlined in Figure 1B, these two promoters differ in the spacing between tet operators. The center-to-center distance of neighboring operators is 42 nt for Ptet-1 versus 36 nt for Ptet-14. Moreover, their respective CMV minimal promoter moieties differ in length.

The T-series of Ptet promoters introduced here all contain a tet operator heptamer with 36 nt spacing (Figure 1C). In contrast to Ptet-14 where the spacer sequences between the operators are identical we used randomized spacer sequences (additional file 1), thus minimizing the symmetry within the operator region. This accounts for the sensitivity of viral vectors to palindromic sequences due to the strand displacement mechanism [21,22], which may lead to gradual loss of operators during reverse transcription [17,23]. Replacing the operator heptamer of Ptet-1 with this newly designed operator array resulted in Ptet-T1.

Subsequent modifications of the minimal promoter moiety of the Ptet promoters followed a rational design strategy, stepwise altering known functional sequence elements within the CMV minimal promoter as used in Ptet-T1. Introducing point mutations to Ptet-T1 led to consensus sequences of the TATA-box and the TFIIB binding site (BRE [24,25]) in Ptet-T2. The BRE upstream the TATA-box could possibly enhance transcriptional initiation upon induction with acidic domain-type transactivator VP16 [26-29], while reducing background transcription of promoters in the non-induced state [26,30]. Deletion of the CMV 5'-UTR led initially to Ptet-T3 with truncated downstream promoter element (DPE,[31]). Ptet-T4, exhibiting a rudimentary 5'-UTR, contained only the CMV-initiator element (Inr, [32]) directly followed by the start codon. In order to restore an average length 5'-UTR, we decided to introduce an unrelated eukaryotic sequence from a plant RNA virus (turnip yellow mosaic virus, TYMV) fused 3' to the Inr sequence of Ptet-T4. This sequence contains only few *cis *elements known to be important for promoter regulation in the animal kingdom (Transfac database; http://www.gene-regulation.com/pub/databases.html). The transcription factor binding sites found, for RFX-1 and AP-4, were eliminated by point mutations, resulting in Ptet-T5. Further modifications of the TYMV 5'-UTR led to Ptet-T6 and Ptet-T7. As discussed in more detail below, these constructs are devoid of potential splice signals (5'-AGGT-3' to 5'-AGCT-3'; see additional file 2) and hairpin structures [33,34] that could confer cap-independent translation initiation.

We were primarily interested in functional properties of the newly designed Ptet promoters in heterogeneous, non-clonal cell populations. We therefore evaluated these promoters by transient transfection of plasmid DNA, as well as γ-retroviral transduction resulting in stable cell pools.

### Initial characterization of the new promoters in a plasmid backbone

**Figure 2:**
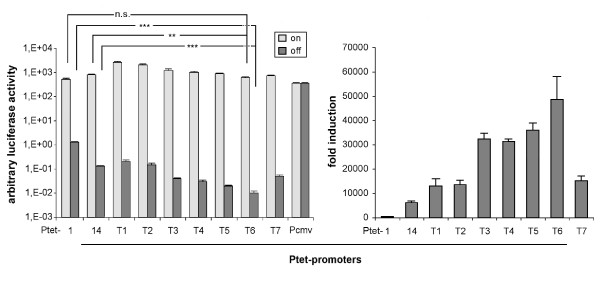
**Transient transfection analysis of the new Ptet promoters in HeLa-EM2 cells**. *Left panel*: Firefly luciferase activities after transfection of HeLa-EM2 cells with the new Ptet promoter driven reporter constructs were determined in the on-(+Dox) and off-state (-Dox) of the system. Values were normalized to the activity of a cotransfected internal standard (beta-galactosidase reporter). Statistical comparison of Ptet-1 and Ptet-14 with Ptet-T6 via students t-test was performed with Graph Pad Prism version 5.03 software (** = p-value < 0,01, *** = p-value < 0.001, n.s. = not significant). High amounts of luciferase reporter plasmids were transfected to achieve relative light unit readings above instrument background that could be reliably quantified. For example, in this experiment the instrument background (identical to mock transfected cells) was 1,5 × 10^2 ^rlu while that of extracts from Ptet-T6 transfected cells (-Dox) was 3,5 × 10^2 ^rlu. *Right panel*: Regulation factors for the individual Ptet promoters from the analysis shown left. The results shown are the mean of three independent transfection experiments, the error bars represent the SEM.

The new Ptet promoters as well as the control constructs (full length CMVie promoter (-673/+75), Ptet-1 and Ptet-14) were all integrated in an identical manner into the plasmid pUHC-131-1 [35] containing firefly luciferase as the reporter gene. Transfection into tet transactivator-positive HeLa-EM2 cells [19] was performed in the presence and absence of Dox. The results as shown in Figure 2 demonstrate that all the new Ptet promoters were highly inducible. Ptet-T1 displayed a 5-fold higher induced expression level than Ptet-1, although both contain the identical CMV minimal promoter. Ptet-T1 also displayed a 6-fold reduction of non-induced background expression resulting in a regulation factor of at least 13000-fold vs. 400-fold for the original Ptet-1. This improvement is solely based on the new operator heptamer introduced into otherwise identical constructs. The expression characteristics of Ptet-T2 were similar to that of Ptet-T1, differing only by the introduction of a consensus BRE in the T2 construct.

Modifications of the 5'-UTR (constructs Ptet-T3 to-T7) led to slightly reduced expression in the induced state when compared to Ptet-T2. Still, even the weakest of these Ptet promoters, Ptet-T6, displayed a slightly higher induced expression level than the original Ptet-1.

In line with our experimental objective, the more interesting outcome of the stepwise alteration of the minimal promoter when modifying the 5'-UTR was the further reduction of background expression in the off-state, already obvious from shortening the CMV leader sequence in Ptet-T3 and Ptet-T4 (> 30-fold reduction when compared to Ptet-1). Introduction of the TYMV 5'-UTR and its variants (Ptet-T5, -T6 and -T7) reduced background expression even further. Especially Ptet-T6 displayed significantly lower background activity, more than 100-fold reduced compared to Ptet-1 and about 10-fold compared to Ptet-14. This resulted in regulation factors of about 50000-fold in these transient transfection assays (Ptet-1 = 400-fold; Ptet-14 = 6200-fold).

It should be noted here that for several of the Ptet promoters the luciferase values measured by us were very close to instrument background. Compared to our established protocols we increased the amount of transfected plasmid DNA in order to obtain readings that could be reliably quantified (see Methods and legend of figure 2).

### Performance of the new regulatory units after retroviral transduction

**Figure 3:**
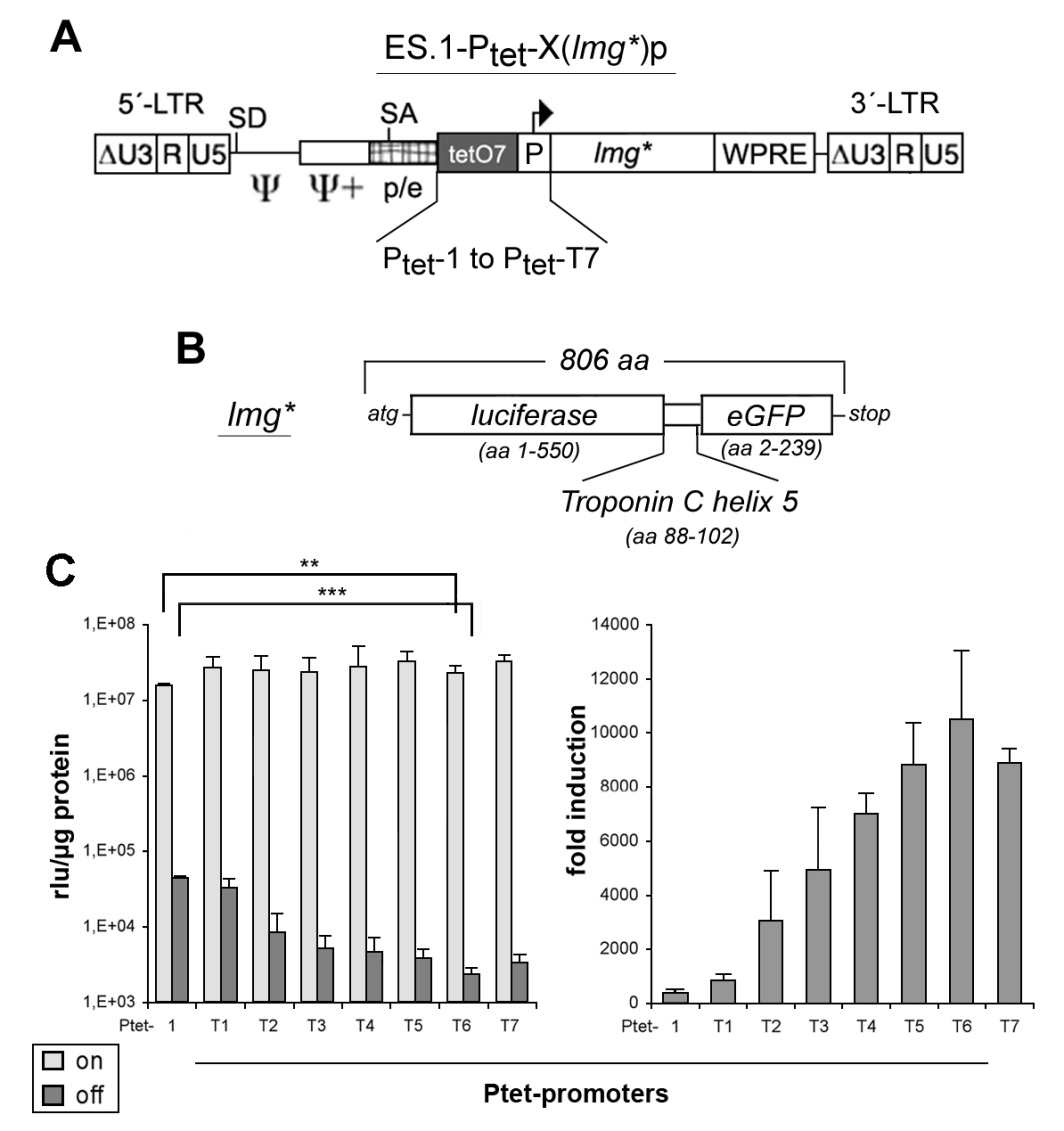
**Retroviral transfer of the new Ptet promoters**. **(A) **The different regulatory units were inserted into the γ-retroviral self-inactivating (SIN) vector „ES.1". The U3-enhancer elements (ΔU3) were deleted from the provirus, while the enlarged packaging region (ψ,ψ+) as well as the native splice acceptor (SA) located in the pol/env region of the virus were retained. In order to enhance viral titers and translational efficiency, a woodchuck posttranscriptional regulatory element (WPRE) was integrated 3'-to the reporter gene. **(B) **The lmg* dual reporter was used throughout all experiments with the viral vectors. The corresponding gene consists of the firefly luciferase open reading frame (orf), with deleted stop codon, the 3'-half of the *troponin C *α-helix5 and the eGFP orf with the deleted start codon. **(C) ***Left panel*: Determination of specific luciferase activity (relative light units, rlu) that were obtained from transduced, FACS enriched HtTA-1 cell populations. Statistical comparison was done as described in figure 2. *Right panel*: Regulation factors for the individual Ptet promoters from the analysis shown left. The results shown are derived from at least two cell populations generated independently. Each population was analyzed two to three times; the error bars represent the SEM.

The newly designed Ptet promoters were integrated into the ES.1 γ-retroviral vector (Figure 3A) containing lmg* as the reporter gene (Figure 3B). Lmg* is a dual reporter gene providing GFP as well as luciferase activity (see Methods). This allowed us to determine differences in unregulated background expression with exceptional sensitivity in luciferase assays without sacrificing the option of single cell resolution via GFP measurements.

Infections of HtTA-1 cells (expressing the tet-off type transactivator tTA; [1]) were performed at low MOI (0.06-0.12) under induced conditions (to allow cell enrichment of all GFP positive transductants). This ensured that the majority of the transduced cells contained a single integrated copy of the response unit [36]. The results shown in Figure 3C were similar but not identical to those of the transfection experiments (Figure 2). All new Ptet promoters displayed high expression levels in the induced state, slightly higher than Ptet-1.

A direct comparison of reporter activities in absolute terms is not possible between the different experiments, as we had to switch from luciferase activity normalized to a second co-transfected reporter (transient transfections; Figure 2) to luciferase activity normalized to the protein content of cellular extracts (stable transductions; Figure 3). However, the pattern of background expression levels in the off-state of the system were similar between the different Ptet promoters when comparing the results shown in Figure 2 with those of Figure 3. As in case of the plasmid transfections the Ptet-T6 transduced cell populations displayed the lowest background expression after chromosomal integration. The only notable difference resulted from the introduction of the BRE (transition from Ptet-T1 to -T2), which had a much more pronounced effect on background expression upon chromosomal integration.

### Comparison of the doxycycline dose response of Ptet-1 and Ptet-T6

**Figure 4:**
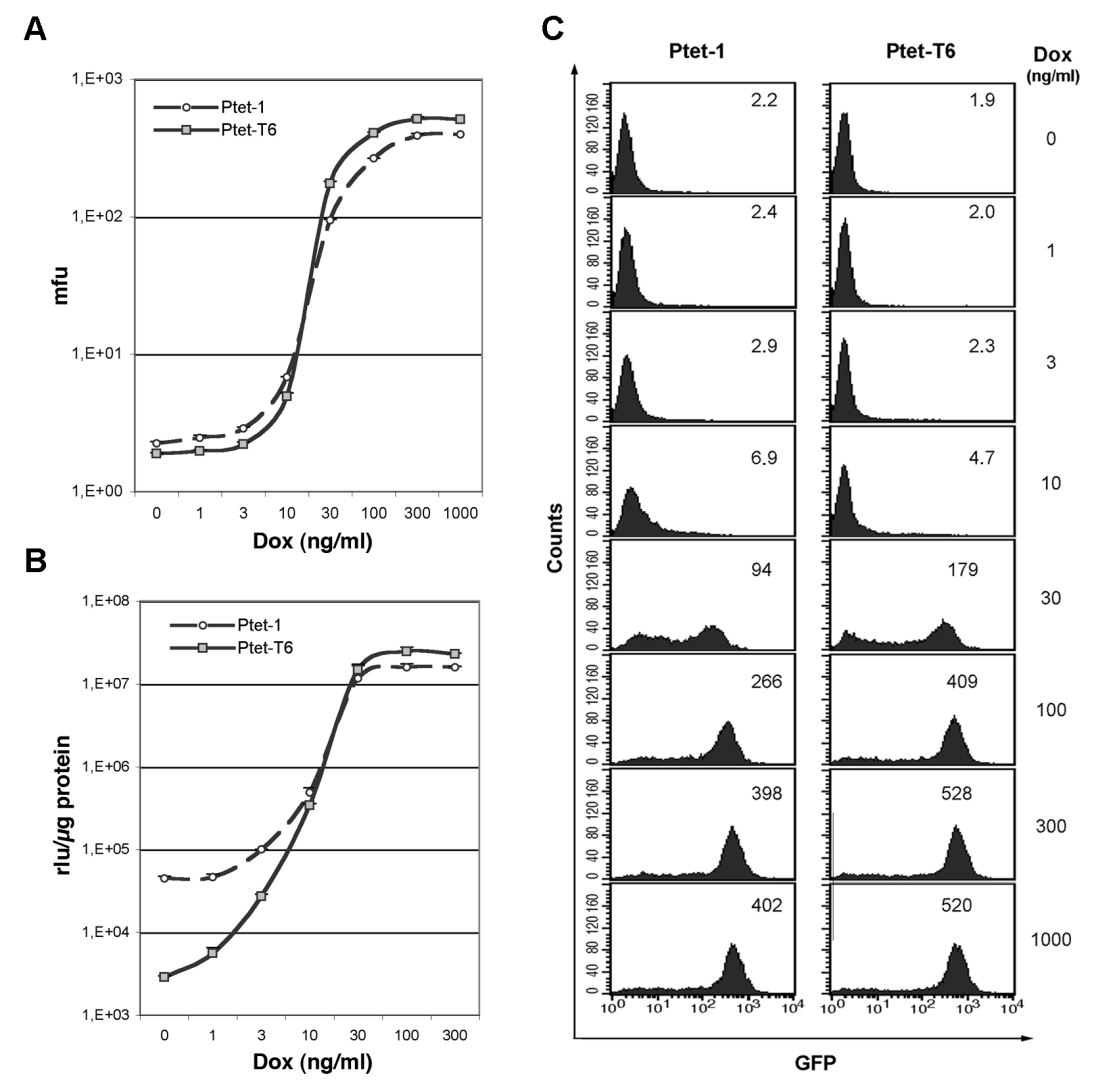
**Dose response analyses of Ptet-1 and Ptet-T6 in transduced HeLa-EM2 cell populations**. **(A) **Determination of dose response via eGFP function of lmg*. Mean fluorescence units (mfu) are given for the complete populations (≥ 95% purity). For value ranges see insets in "C". **(B) **The simultaneous analysis of identical populations for specific luciferase activity of lmg*. The rlu values ranged from about 4.5 × 10^4 ^(± 2.8 × 10^3^) to 1.6 × 10^7 ^(± 4.7 × 10^5^) rlu/μg for Ptet-1 and 2.9 × 10^3 ^(± 1.1 × 10^2^) to 2.3 × 10^7 ^(± 3.9 × 10^5^) rlu/μg for Ptet-T6 in the off-and on-state of the system. **(C) **FACS-analysis of a representative cell population for Ptet-1 and Ptet-T6 promoters transferred by the viral vectors. Mfu values of the whole population were given as inset. The cellular background of the GFP-negative Hela-EM2 parental cell line was 1.83. The Dox concentrations used are indicated. Induction was for 96 hours. Results shown in figs. A and B were obtained from two independently generated populations for each vector.

Next, we asked whether the altered Ptet promoter architecture would result in dose response changes of the regulatory system towards Dox. These parameters were previously established for Ptet-1 in HeLa cells. HeLa-EM2 cells were transduced by ES.1-Ptet-1(lmg*)p or ES.1-Ptet-T6(lmg*)p retroviral SIN-vectors. Purified populations were incubated in the presence of the indicated Dox concentration for 96 hours, harvested and analyzed for eGFP fluorescence and luciferase actvity (Figure 4A and 4B). The GFP data confirm that Ptet-T6 can reach a slightly improved expression level when compared to Ptet-1. Since in the non-induced state, the mean fluorescence of both cell populations is too close to the background level of HeLa-EM2 cells no meaningful conclusion (Figure 4A) can be drawn.

Taking advantage of the luciferase activity of the lmg* reporter, simultaneous luciferase measurements revealed that background expression in the off-state of Ptet-T6 is 15-fold below that of Ptet-1, in line with the previous analysis (Figure 4B). The combination of reduced background and slightly enhanced induced expression level resulted in an about 30-fold increased regulation range. The maximum induction for both promoters had been reached at 300 ng Dox/ml; further increase of the effector concentration was without effect.

Note that the steep increase of gene expression between 10 and 100 ng Dox/ml is similar for both Ptet promoters, indicating an identical synergistic response of both promoters to increased occupancy of the operators by tet-transactivators.

The FACS analysis at single cell resolution (Figure 4C) indicates that all cells responded to induction, but not homogenously. Upon partial induction (30 ng Dox/ml) the expression level of the individual cells differed widely. It has to be emphasized that the cell population analysis was genetically heterogeneous with respect to insertion of the reporter unit. Again, the overall characterization of the two Ptet promoters analyzed revealed no major differences in the dose response parameters.

### Fine-tuning of transgene expression

**Figure 5:**
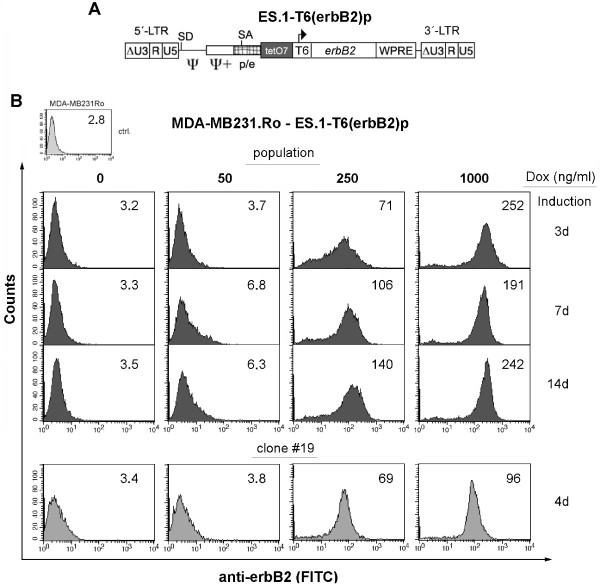
**Transduction of a Ptet-T6 regulated erbB2 expression unit into MDA-MB231.Ro cells**. **(A) **γ-retroviral SIN-vector used to transfer erbB2 into rtTA-S2-positive MDA-MB231.Ro target cells. **(B) **FACS-analysis of transduced cell populations induced for the indicated time and Dox concentrations. The expression of the erbB2 transgene was detected by a monoclonal antibody (mAB ALX-804-573; Alexis) in combination with a fluorescence labeled secondary antibody (donkey anti mouse IgG-FITC, affipure, Jackson Immuno Res.). Mean fluorescence units (mfu, blot inset) are given for the complete population (≥ 92% purity). Clone #19 was derived from this population by limiting dilution.

The results obtained with the new Ptet promoters, especially Ptet-T6, led us to routinely employ these promoters in ongoing research projects in our laboratories. The following results were derived from a project aiming at the conditional expression of erbB2 in breast cancer cell lines. A tet transactivator-positive MDA-MB231.ro cell line expressing rtTA-S2 [6] has been previously established (R.L., unpublished results). Transduction of this clone was performed with a retroviral SIN-vector, ES.1-T6(erbB2)p (Figure 5A), in which the *erbB2 *(or Her2/neu, [37,38]) open reading frame was under control of Ptet-T6. As the ErbB2 protein is located on the cell surface, it is well suited for detection and selection of the transduced population by FACS.

The results of the cell-based analysis (Figure 5B, triple panel) of the purified population showed, that (i) erbB2 expression was tightly regulated in these experiments with no recognizable background expression in the off-state, (ii) despite looking at non-clonal pools expression could be adjusted to intermediate levels by titrating Dox, and (iii) cells could be maintained at a chosen expression level over a prolonged period of time. This time course analysis was terminated on day 14. Interestingly, prolonged full induction (1000 ng Dox/ml) led to the disappearance of erbB2 cells, showing highest expression levels, as can be seen from the asymmetric steep drop of the right shoulder of the expression profile peak. Thus, the expression level appears to reflect the maximum long-term expression level of erbB2 tolerated by those cells. The only other recognizable difference in the expression profiles was a slight „compression" of intermediate expression at 250 ng/ml Dox, as the cell-to-cell variability in transgene expression was apparently reduced after prolonged growth under these conditions. Given the non-clonal origin of this cell population, individual cells behaved remarkably similar. Still, for certain applications, like analyzing pharmacological responses to varying densities of cell surface receptors, the homogeneity under intermediate expression conditions may not be sufficient for reliable results. Thus, we examined whether respective homogeneous cell populations, derived from cell clones, would improve the situation. The example shown in Figure 5B, lower panel, demonstrates that this is indeed the case. Further suppression of background expression could already be observed in the vast majority of cells, but the precision of partial induction was markedly improved by clonal isolation of cells harboring Ptet-T6.

## Discussion

Our goal was to improve the dynamic range of tetracycline regulated transcription in such a way that tight, virtually background-free non-induced expression can routinely be obtained when working with non-clonal cell populations. The Tet system in its current configuration has proven its capacity to achieve such background free expression, as widely documented in the literature. However, most of these studies relied on clonal analysis of transgenic cells, thereby eliminating cell clones with less favorable properties, an often laborious process which at times might even be impractical, for example in the genetic engineering of primary cells. By using previously established and well-characterized transactivator-positive cell lines we restricted our efforts solely to *cis *elements of tet-controlled transcription units, the tet-responsive promoters. We investigated if gene expression regulated by a rationally designed series of new Ptet promoters would be superior over the widely used original Ptet-1 promoter and its derivative Ptet-14 (P_tight_), particular by further reducing background expression of tet-regulated transcription units while maintaining fully-induced expression levels. We conclude that the reduction of the Ptet background expression reported here is exclusively due to the low intrinsic background of these new tet-responsive promoters. Their side-by-side analysis (together with the previously characterized Ptet-1 and Ptet-14) in different established tet transactivator-positive cell lines eliminated the possibility that effects like transactivator abundance or differences in tTA-vs. rtTA-mediated transcription control would influence our conclusions. Moreover, the tet operator sequences themselves are identical in all new Ptet promoters used and any potential residual transactivator affinity to the operators would not have any influence on the outcome of our study. Lastly, we excluded a systematic distortion of our results by the possible occurence of integration site-specific or epigenetic effects through parallel analysis of all Ptet promoters in transient transfection assays.

The initial transition from Ptet-1 to Ptet-T1 resulted in a more than 5-fold lower background expression in transient transfection assays. This effect was less pronounced after viral transduction, possibly somewhat overridden by the preference of γ-retroviral vectors for promoter-proximal integration [39] and the resulting elevated background expression levels. In any case, the improvements observed are solely due to the newly designed tet operator heptamer with 36 nt spacing between the centers, as the hCMV minimal promoter (-53/+75) remained unaltered. So far, the relative contribution of spacer length variations versus alterations of nucleotide sequences separating them remains unclear. Changes in the phasing of bound transactivators cannot account for different background expression levels, only for differences in activated expression. It is interesting to note, that for the available 36 nt spaced operator constructs ([13]; this study) varying induction levels were obtained compared to a 42 nt operator spacing in the context of otherwise identical promoters. In contrast to our own results (Ptet-1 vs. Ptet-T1) showing an increased inducible expression level with the 36 nt spaced operators, the study of Agha-Mohammadi showed a reduction in induced expression ([13]; pCMV*-2 vs. p8tetO-36). So far we do not have an explanation for these differences.

The first step towards improving the regulatory properties of the CMV minimal promoter was the introduction of a high affinity TFIIB binding site (BRE, [25]) upstream of a consensus TATA-box into Ptet-T1. This, other than the changes in the operator heptamer, led to a reduction of background activity most pronounced after stable integration of the Ptet-T2 by a retroviral vector. These results are in line with published data that implicate a negative effect on basal transcription levels for such promoter modifications without affecting the activated levels of transcription [26]. In addition, the composition of minimal promoters with respect to *cis *elements or the interaction of different acidic activation domains with TFIIB may play a significant role in the process of non-induced and induced transcription, and might be modulated depending on the transacting factors available in a given cell type [30,40,41].

To further reduce the unregulated background expression we truncated the 5'-UTR of the CMV-minimal promoter. Deletions of promoter elements within the leader, e.g. the DPE, have been shown to improve the signal to noise ratio of the minimal promoter [13,18]. Indeed, a direct comparison of Ptet-14, Ptet-T3 and Ptet-T4 with Ptet-1 by transfection, revealed a strongly reduced background expression in the off-state for these three promoters (10-, 30-and 40-fold, respectively). Thus, in the transient state the expected reduction of background expression by the BRE became functional only after removal of the DPE element [31]. A pronounced reduction of baseline expression of Ptet-T3 and Ptet-T4 vs. Ptet-14 suggests that the suppressing effect of TFIIB on background transcription was partly masked by the DPE element. However, the effect of deleting the DPE might not be directly related to the function of the BRE.

In case of Ptet-T4 we relied on an unusually short 5'-UTR. Given the importance of untranslated leader regions for the functionality of mRNAs and reconciling their average length of around 200 nt [42] we tried to increase their length without losing the gains in dynamic range. In order to increase the size of the untranslated leader, a 5'-UTR fragment of the Turnip Yellow Mosaic virus (TYMV, [34]), was fused downstream to the residual CMV sequence of Ptet-T4. This leader was chosen because we assumed that *cis*-elements of the plant kingdom will not or only by chance be effective in the mammalian background. Indeed, the unregulated background expression was further reduced by Ptet-T5. The elimination of two hairpins within the TYMV 5'-UTR fragment [33] resulted in further reduction of background expression in the off-state of the Tet-system. This holds true for both, the transient and the stable approach, since Ptet-T6 displayed a 50000-fold up-regulation of gene expression in the plasmid based system (Figure 2) and about 10000-fold after its stable integration by a γ-retroviral vector (Figure 3).

Among all the different promoter designs tested, Ptet-T6 was consistently found to be the optimal Ptet promoter. We are currently using this promoter routinely in our laboratories when setting up tet-responsive stable cell lines. Ptet-T6 promoter has been successfully tested in stable cell lines as diverse as HEK293, primary human fibroblasts and an avian cell line (MG, unpublished results). Here we also showed that the Dox dose response of this promoter was unaltered when compared to the original Ptet-1, allowing an efficient, titratable expression control even in transfected cell populations. In many applications these improvements will allow tight control over transgene expression while omitting the time consuming step of clonal isolation. However, our results for the comparative analysis of pooled vs. clonal isolates of erbB2 expressing cells indicated that, obviously, for the precise adjustment of intermediate level expression the pool strategy is not optimal. Indeed, pools showed rather heterogeneous expression levels under these induction conditions, most likely due to integration site effects with individual loci following their inherent partial induction schemes. This would be expected from differences in e.g. chromatin packaging. Considering the different causes responsible for background expression in the off-state, such effects have to be addressed by means other than optimizing Ptet promoters for their lowest level of intrinsic background expression.

## Conclusion

This study demonstrated the feasibility to further improve tetracycline responsive promoters, especially for reduction of background expression of the Tet regulatory system in the off-state. To confirm that the newly created Ptet design is of general use, the promoters were analyzed in two different assay systems, namely following transient transfection or otherwise chromosomal integration. Given our limited understanding of many aspects of mammalian promoter architecture, modifications of *cis *elements will always be partly empirical. The results presented here nevertheless show, that some of the promoter elements tested, display similar behavior whether analyzed in the context of Ptet promoters or in their original promoter environment. From our previous experience we are confident that these findings from different cell culture systems will hold up in animal experiments, although this has yet to be proven. Thus, we anticipate that these novel Ptet promoters will contribute to further refining and enhancing the Tet technology for *in vivo *and *in vitro *use.

## Methods

### Cell culture

293T cells (ATCC # CRL-11268) were cultured in Dulbecco's modified Eagles medium (DMEM, Invitrogen) supplemented with 10% heat inactivated fetal calf serum (FCS, PAA) at 37°C, 5% CO_2_. HtTA-1 [1] and HeLa-EM2 cells [19] were cultured in Modified Eagles Medium (MEM, Invitrogen) supplemented with 10% heat inactivated FCS at 37°C, 5% CO_2_. The HtTA-1 cell line expressed the originally described tet-responsive transactivator tTA, active in the absence of Dox (Tet-Off), while HeLa-EM2 cells express the codon optimized M2-reverse transactivator [6] in the cellular background which is fully active around 300 ng Dox/ml (Tet-On).

The MDA-MB231 cells, a human breast cancer cell line (ATCC #HTB-26), modified to constitutively express the S2-reverse transactivator, termed MDA-MB231.Ro. rtTA-S2 is an alternative codon-optimized reverse transactivator, that is fully active in the presence of 1000 ng Dox/ml [6]. Cultivation is performed at 37°C without CO_2 _in Leibovitz L-15 medium (Invitrogen, #11415-049), supplemented with 10% FCS and 2 mM L-glutamine.

Cultures were split at 70-80% confluence. Cells were harvested after washing with PBS by incubating for 3-5 min with PBS/EDTA (0,8 mM), before being transferred into fresh medium or being used for analysis.

### Transient vector production and titration

Transient production of viral vectors was carried out essentially as described earlier [17]. Briefly, about 1.5 × 10^6 ^293T cells were transferred to 60 mm dishes the day before transfection. A total amount of 15 μg plasmid DNA with 5 μg each of pHIT60 (gag/pol expression plasmid; [43]), of pczVSV-G (VSV-G envelope expression plasmid; [44]) and of the transfer vector was transfected via lipofection with the TransIt293 reagent (Mirus, CA) as recommended by the supplier. 16-18 hours after transfection the cells were incubated in medium supplemented with Na-butyrate (5 mM) for 6-8 hours. The culture supernatant was harvested 16-18 hours following medium exchange, filtrated (0.45 μm) supplemented with polybrene (8 μg/ml, SIGMA), aliquoted and stored at -80°C for later use.

All titrations were performed on HtTA-1 cells via serial 2-fold dilution of supernatants (5-10-20-40-80-160-fold) containing infectious particles (IP). Briefly, 2 × 10^5 ^cells were transferred to a 6-well dish the day before infection. 24 hours later, medium was replaced by 1 ml fresh culture medium, premixed with vector containing supernatant. About 16-20 hours later the incubation medium was replaced by fresh medium and cells were cultivated under induced conditions. FACS analysis was performed about 96 hours post transduction. Vector titers were determined according to the number of GFP positive cells (4 × 10^5 ^cells × % GFP-pos/100), multiplied with the dilution factor as well as a correction factor 2 to account for cell division during infection. The titers for all vectors described in this work were in the range of 1-3 × 10^6 ^infectious particles (IP)/ml.

### Establishing transduced cell populations

About 4 × 10^5 ^cells (HtTA-1, HeLa-EM2 or MDA-MB231.Ro) were infected with serial dilutions of the transiently produced vectors and induced for four to five days with (1000 ng Dox/ml, HeLa-EM2, MDA-MB231.Ro) or without Dox (HtTA-1). All GFP-positive cells of appropriate infected cell populations (1-3% positive to ensure mostly single copy integrates) were used for the enrichment by fluorescence activated cell sorting (FACS). Pool sizes were adjusted to always exhibit > 10000 independent clones by multiple independent infections. For each of the Ptet promoter driven reporter constructs populations of ≥ 90% purity were used throughout the experiments.

### Determination of luciferase activity

Transient transfection by lipofection and reporter assays for Ptet promoter driven firefly luciferase and CMV promoter driven beta-galactosidase (internal transfection control) were performed as described [45]. For each well of a 6-well plate 0.5 ug of luciferase reporter had to be transfected to obtain reliable readings for non-induced cultures. The luciferase activity of HeLa-EM2 and HtTA-1 cells was indistinguishable from the technical background in untransfected populations.

Purified transduced cell populations were cultivated in the off-state for at least 10 days. This is necessary because of the prolonged half life of luciferase in the fusion protein lmg* and the high expression levels reached in the induced state (see below). Induction experiments were started by splitting about 1 × 10^5 ^off-state cells into cell culture medium with (500-1000 ng/ml) or without Dox. After 96 hours the cells were harvested with PBS/EDTA and used for analysis of fluorescence and luciferase activity in the on-and off-state. The lyses of the cells and treatment of samples was essentially as described [46]. Specific luciferase activity was calculated after determining protein concentration of the samples by the method of Bradford [47]. Treatment of cells was similar for the dose response experiments, except that here the medium was exchanged daily (supplemented with the indicated Dox-concentrations) as a precautionary measure to account for the possible degradation of Dox at very low concentrations.

### Synthesis of the dual reporter gene: lmg*

To facilitate the simultaneous determination of reporter activity in cell populations as well as in individual cells contained herein, we generated a fusion protein consisting of firefly luciferase and eGFP.

The reporter gene fusion was generated by introducing a fragment coding for an α-helical spacer (troponinC, helix 5, see Figure 3B) between a 5'-luciferase (deleted stop codon) and a 3'-eGFP (deleted start codon) open reading frame (orf). The whole frame was synthesized by overlapping PCR. Briefly, using pUHC-131-1 [35] as template for luciferase and SK-eGFP as template for the eGFP coding region, two PCR fragments were synthesized that served as template for a second PCR reaction delivering the final orf. The deletion of the luciferase stop codon and the 5'-part of the helical region was introduced into the 3'-oligo of luciferase while the 3'-part of the helical region and the deletion of the eGFP start codon was contained within the 5'-oligo of eGFP (additional File 3). The dual reporter gene has most useful properties as had been determined in HtTA-1 cells: (i) luciferase combined half-life is expanded to about 9 hours (~3 ×longer compared to wildtype luciferase; [48]), and (ii) eGFP half life was reduced to about 11 hours (~3 × shorter compared to eGFP alone, [49]). The half-life of both reporter gene activities was determined in HtTA-1 cells transduced by ES.1-Ptet-T6. The enriched population was induced for 96 hours. Induction was stopped by addition of Dox to the culture medium. Cells were taken directly from the induced population and measured in FACS (mean fluorescence intensity) or otherwise lysed for luciferase activity determination. The lysates were stored at -20°C for later analysis. The remaining cells were further cultivated in medium with Dox (off-state) and samples were harvested as described above at 1, 2, 4, 8, 12, 24 and 48 hours. Linear regression analysis was employed to determine the time point within the linear range of decreasing activity at which half of the activity was lost.

Unlike similar approaches to generate dual reporter genes, using the renilla luciferase and aeqourea GFP ([50]), no energy transfer is expected between the two separated entities (emission luciferin 569 nm, excitation/emission eGFP 488/509 nm, [51]). Thus, distinct measurements of a highly sensitive luciferase activity and a more dynamic eGFP are possible.

### Plasmid constructs

The Ptet promoter units were constructed as exchangeable modules in the pBluescript SKII+ plasmid backbone (Stratagene). All cloning and modification steps were performed according to established standard procedures [52] or as recommended by the suppliers. Synthesis of the tet operator heptamer was performed by subsequent annealing/ligation steps of pre-annealed ds-oligos, providing individual overhangs and the spacers with random nucleotide sequences between the operators. 5' XhoI and 3' Hind III restriction sites were also included in the heptamer design (additional File 1). A CMV-minimal promoter with 3' SalI and NcoI restriction sites was introduced 3' of the heptamer. This synthetic fragment, termed TO7.3, was cloned as XhoI/NcoI fragment into similar digested pBluescript SKII+ into which the orf of eGFP had already been introduced (5' EcoRI/NcoI and 3' NotI), creating SK-TO7.3g. EGFP was exchanged for lmg* (NcoI/NotI) leading to SK-TO7.3lmg* which served as basis for insertion of all promoter variants of the Ptet-T series.

The promoter variants were either PCR amplified using particular sense and antisense oligonucleotides or generated as variants via site directed mutagenesis by standard techniques, subcloned into pBluescript SKII+ and sequenced. The T1-T7 minimal promoters (additional file. 2) were introduced as HindIII/SalI fragments into SK-TO7.3-lmg*, resulting in SK-Ptet-T1, -Ptet-T2, -Ptet-T3, -Ptet-T4, -Ptet-T5, -Ptet-T6, and Ptet-T7.

Similar, the original Ptet-1 promoter [1], containing the 42nt spaced tet operators and a CMV-minimal promoter (-53/+75), was released as XhoI/NcoI from S2f-c(LCMG) [17] and inserted into SK-TO7.3-lmg*, resulting in SK-Ptet-1.

The Ptet promoters were transferred as XhoI/NcoI fragments into the pUHC-131 expression vector [35] which contains the firefly luciferase reporter.

The retroviral SIN-vector "pES.1" used for the transfer of the tet-response units had been described earlier [46]. Individual inducible expression cassettes, consisting of the tet operator heptamer, a minimal promoter, the lmg* reporter gene were integrated as XhoI/NotI fragments (released from respective SK-TO7."x"-lmg* plasmids) 5' to the posttranscriptional regulatory element (WPRE) of the woodchuck hepatitis virus [53]. Transcription of the pES.1-Ptet-1 and pES.1-Ptet-T1 to Ptet-T7 vectors is terminated at the polyadenylation signal of the viral 3'-LTR.

Thus, irrespective of the plasmid or viral vector used for transfer of the regulatory units, the surrounding sequences were kept constant to rule out the possibility of misleading results caused by unpredictable effects of different spacing or altered sequence elements.

## Competing interests

RL and HB have filed a patent application covering the promoters described in this manuscript (assignee: TET Systems GmbH & Co.KG, Heidelberg). MG and HB acknowledge financial interest in TET Systems GmbH & Co.KG, Heidelberg.

## Authors' contributions

RL designed and cloned the Ptet promoters, participated in the viral work, the analysis of data and drafted the manuscript. NH participated in viral vector cloning and their functional analysis. MH did all plasmid based experiments. HB co-designed the study and wrote the manuscript. MG co-designed the study, analysed the plasmid data, and wrote the manuscript. All authors read and approved the final version of the manuscript.

## Supplementary Material

Additional file 1 **Tet operator heptamer of the Ptet-T series of Ptet promoters**. The identical tet operator sequences are underlined, their imperfect inverted repeats are shown in lower case. The center-to-center distance between neighbouring operators is 36 nt, the respective centers are capitalized and bold. Unique restriction sites used were 5'- XhoI and 3'- Hin dIII. Click here for file

Additional file 2 **Minimal promoters**. Upstream (upper panel) and downstream promoter sequences (lower panel) relative to the transcription start site (+1) are aligned separately. 5' (Hin dIII) and 3' (Sal I) restriction sites are shown in lower case, bold. The CMV-initiator (Inr) is underlined. TATA-Box and TFIIB site are shaded in grey. The 5'-UTRs are in lower case letters, for the TYMV 5'-UTR derived constructs also in italics. Note that the Ptet-1 (not shown) and Ptet-T1 minimal promoter sequences are identical, these two Ptet promoters differ only in their tet operator array. Click here for file

Additional file 3 Amino acid sequence of lmg* dual reporter. The firefly luciferase orf is from aa 1-546 (upper case letters), the tropnonin C spacer from aa 547-567 (underlined), and the eGFP-orf from aa 568-807 (lower case letters). The last amino acid of the original luciferase orf (a leucine) together with the stop codon has been removed, as well as the original start codon of the eGFP-orf. Click here for file
