# Supplementary material for: Improved Tet-responsive promoters with minimized background expression
Source: BMC Biotechnol. 2010 Nov 24;10:81. doi: 10.1186/1472-6750-10-81 (PMC3002914; doi:10.1186/1472-6750-10-81)
Supplement: Additional file 1 — Tet operator heptamer of the Ptet-T series of Ptet promoters. The identical tet operator sequences are underlined, their imperfect inverted repeats are shown in lower case. The center-to-center distance between neighbouring operators is 36 nt, the respective centers are capitalized and bold. Unique restriction sites used were 5'- XhoI and 3'- Hin dIII. [file 1472-6750-10-81-S1.DOC]

**XhoI**

**ctcgag**TTTACtccctatca**G**tgatagagaACGTATGAAGAGTTTACtccctatca**G**tgatagagaACGTATGCAGACTTTACtccctatca**G**tgatagagaACGTATAAGGAGTTTACtccctatca**G**tgatagagaACGTATGACCAGTTTACtccctatca**G**tgatagagaACGTATCTACAGTTTACtccctatca**G**tgatagagaACGTATATCCAGTTTACtccctatca**G**tgatagagaACGTAT**aagctt**

**HindIII**
