# Supplementary material for: Improved Tet-responsive promoters with minimized background expression
Source: BMC Biotechnol. 2010 Nov 24;10:81. doi: 10.1186/1472-6750-10-81 (PMC3002914; doi:10.1186/1472-6750-10-81)
Supplement: Additional file 2 — Minimal promoters. Upstream (upper panel) and downstream promoter sequences (lower panel) relative to the transcription start site (+1) are aligned separately. 5' (Hin dIII) and 3' (Sal I) restriction sites are shown in lower case, bold. The CMV-initiator (Inr) is underlined. TATA-Box and TFIIB site are shaded in grey. The 5'-UTRs are in lower case letters, for the TYMV 5'-UTR derived constructs also in italics. Note that the Ptet-1 (not shown) and Ptet-T1 minimal promoter sequences are identical, these two Ptet promoters differ only in their tet operator array. [file 1472-6750-10-81-S2.DOC]

**Ptet-T** minimal promoter series:

Hin dIII TFIIB TATA Inr

-52 **-30 -23** **+1**

**T1** **aagctt---**GGTAGGCGTGTACGGTGGGAGGCCtatataaGCAGAGCTCGTTTAGTGAACCG**t**cagatcgcctggag

**T2**  **-**GGTAGGCGTGTACGGTggg**c-**gcctata**a**aaGCAGAGCTCGTTTAGTGAACCG**t**cagatcgcctggag

**T3** ---TAGGCGTGTACGGTggg**c-**gcctata**a**aaGCAGAGCTCGTTTAGTGAACCG**t**cagatcgcctggag

**T4** ---TAGGCGTGTACGGTggg**c-**gcctata**a**aaGCAGAGCTCGTTTAGTGAACCG**t**cagatcgcctggag

**T5** ---TAGGCGTGTACGGTggg**c-**gcctata**a**aaGCAGAGCTCGTTTAGTGAACCG**t**cagatcgcctggag

**T6** ---TAGGCGTGTACGGTggg**c-**gcctata**a**aaGCAGAGCTCGTTTAGTGAACCG**t**cagatcgcctggag

**T7** ---TAGGCGTGTACGGTggg**c-**gcctata**a**aaGCAGAGCTCGTTTAGTGAACCG**t**cagatcgcctggag

Common to all 3´-ends:

Inr +75 Sal I Nco I

**T1** CG**t**cagatcgcctggagacgccatccacGCTGTTTTGACCTCCATAGAAGACACCGGGACCGATCCAGCCTCCGCG--- **gtcgac**A**ccatgg**

**T2** CG**t**cagatcgcctggagacgccatccacGCTGTTTTGACCTCCATAGAAGACACCGGGACCGATCCAGCCTCCGCG

**T3** CG**t**cagatcgcctggagacgccatccacGCTGTTT------CCATAGAAGA

**T4** CG**t**cagatcgcctggag

**T5** CG**t**cagatcgcctggag*gtaatcaactaccaattccagctctcttttgacaactggtcttataccaactttccgtaccactt****CCT****accctcgtaagacaattgcaa* **+104**

**T6** CG**t**cagatcgcctggag-------------*aattcca****CAA****c****A****cttttg*--------*tcttataccaactttccgtaccactt****CCT****accctcg****A****aa* **+73**

**T7** CG**t**cagatcgcctggag***C****taatcaactaccaattccagctctcttttgacaactggtcttataccaactttccgtaccactt****CCT****accctc****C****taagacaattgcaaa* **+105**
