# Supplementary material for: Improved Tet-responsive promoters with minimized background expression
Source: BMC Biotechnol. 2010 Nov 24;10:81. doi: 10.1186/1472-6750-10-81 (PMC3002914; doi:10.1186/1472-6750-10-81)
Supplement: Additional file 3 — Amino acid sequence of lmg* dual reporter. The firefly luciferase orf is from aa 1-546 (upper case letters), the tropnonin C spacer from aa 547-567 (underlined), and the eGFP-orf from aa 568-807 (lower case letters). The last amino acid of the original luciferase orf (a leucine) together with the stop codon has been removed, as well as the original start codon of the eGFP-orf. [file 1472-6750-10-81-S3.DOC]

| 10 | 20 | 30 | 40 | 50

1 MEDAKNIKKG PAPFYPLEDG TAGEQLHKAM KRYALVPGTI AFTDAHIEVD

51 ITYAEYFEMS VRLAEAMKRY GLNTNHRIVV CSENSLQFFM PVLGALFIGV

101 AVAPANDIYN ERELLNSMGI SQPTVVFVSK KGLQKILNVQ KKLPIIQKII

151 IMDSKTDYQG FQSMYTFVTS HLPPGFNEYD FVPESFDRDK TIALIMNSSG

201 STGLPKGVAL PHRTACVRFS HARDPIFGNQ IIPDTAILSV VPFHHGFGMF

251 TTLGYLICGF RVVLMYRFEE ELFLRSLQDY KIQSALLVPT LFSFFAKSTL

301 IDKYDLSNLH EIASGGAPLS KEVGEAVAKR FHLPGIRQGY GLTETTSAIL

351 ITPEGDDKPG AVGKVVPFFE AKVVDLDTGK TLGVNQRGEL CVRGPMIMSG

401 YVNNPEATNA LIDKDGWLHS GDIAYWDEDE HFFIVDRLKS LIKYKGYQVA

451 PAELESILLQ HPNIFDAGVA GLPDDDAGEL PAAVVVLEHG KTMTEKEIVD

501 YVASQVTTAK KLRGGVVFVD EVPKGLTGKL DARKIREILI KAKKGGK*IAV*

551 *AKGKSEEELA NCFRIPP*lvs kgeelftgvv pilveldgdv nghkfsvsge

601 gegdatygkl tlkficttgk lpvpwptlvt tltygvqcfs rypdhmkqhd

651 ffksampegy vqertiffkd dgnyktraev kfegdtlvnr ielkgidfke

701 dgnilghkle ynynshnvyi madkqkngik vnfkirhnie dgsvqladhy

751 qqntpigdgp vllpdnhyls tqsalskdpn ekrdhmvlle fvtaagitlg

801 mdelyk*
